# Annotated primary scientific literature: A pedagogical tool for undergraduate courses

**DOI:** 10.1371/journal.pbio.3000103

**Published:** 2019-01-09

**Authors:** Matthew Kararo, Melissa McCartney

**Affiliations:** 1 Department of Biological Sciences, Florida International University, Miami, Florida, United States of America; 2 STEM Transformation Institute, Florida International University, Miami, Florida, United States of America

## Abstract

Annotated primary scientific literature is a teaching and learning resource that provides scaffolding for undergraduate students acculturating to the authentic scientific practice of obtaining and evaluating information through the medium of primary scientific literature. Utilizing annotated primary scientific literature as an integrated pedagogical tool could enable more widespread use of primary scientific literature in undergraduate science classrooms with minimal disruption to existing syllabi. Research is ongoing to determine an optimal implementation protocol, with these preliminary iterations presented here serving as a first look at how students respond to annotated primary scientific literature. The undergraduate biology student participants in our study did not, in general, have an abundance of experience reading primary scientific literature; however, they found the annotations useful, especially for vocabulary and graph interpretation. We present here an implementation protocol for using annotated primary literature in the classroom that minimizes the use of valuable classroom time and requires no additional pedagogical training for instructors.

## Abstract 2

This Community Page article presents Science in the Classroom (SitC), a tool for undergraduate educators to introduce their students to primary scientific literature. Annotations scaffold the readers so that authentic scientific practices are explained and recognized for their importance in scientific communications.

## Background

A major output of public research universities is primary scientific literature, in addition to educating students and conferring degrees. It is imperative for researchers and universities to increase the transparency and outreach of the primary research literature they produce. However, most primary scientific literature remains unknown and/or inaccessible to the public, because it is published in journals targeting academics in the same field and is often placed behind journal paywalls [1].

Public research universities also have a responsibility to produce scientifically literate graduates [2,3]. Many students graduate without an understanding of scientific practices and an acculturation to interpreting scientific communication, especially primary scientific literature [4,5]. One way to potentially improve scientific literacy overall and develop specific skills, such as interpreting scientific communication, is to incorporate primary scientific literature into the undergraduate curricula and provide pedagogical tools that may help bridge the divide between everyday language and the language used by experts [6–11].

The study of primary scientific literature as a pedagogical tool in undergraduate biology courses has led to innovative approaches. The most well-known of these may be the Consider, Read, Elucidate the hypotheses, Analyze and interpret the data, and Think of the next Experiment (CREATE) method, in which faculty redesign their existing courses around primary scientific literature in order to provide an intensive and comprehensive analysis of primary scientific literature for undergraduates [6,12–14]. Although this type of a semester-long innovative elective course provided student benefits, adding an entire course to a degree sequence may prove difficult and by definition, does not impact students that choose not to include them in an already credit-crunched plan of study. This credit-crunch is especially prevalent at institutions such as the one in this study, Florida International University (FIU), where any additional credit hours are charged at out-of-state tuition rates. Therefore, it would benefit biology education, and biology as a field of study, to develop innovative ways to utilize primary scientific literature as a pedagogical tool, ideally with a minimal impact to existing plans of study and time investment from course instructors.

A growing body of research shows that less-intensive interventions using primary scientific literature can be valuable and useful in science, technology, engineering, and math (STEM) education, with the greatest amount of research happening at the undergraduate level. Programs include journal clubs, data and figure exploration, and tutorials on how to read primary scientific literature [15–17]. Assessment tools used to evaluate these interventions are equally as diverse, ranging from rubrics to validated surveys [18,19].

### Annotated primary scientific literature

Annotated primary scientific literature is designed to help readers interpret complex science by overlaying additional information on a scientific research article. Preserving the original text and its context is what makes annotated primary scientific research literature unique from other genres that modify or rewrite the original text. This preservation is the key difference between annotated primary scientific literature and adapted primary literature, an approach that takes portions of primary scientific literature and rewrites the original content to turn them into pedagogical tools [20]. Science in the Classroom (SitC; www.scienceintheclassroom.org) is a highly developed and sophisticated example of annotated primary scientific literature that we decided has potential for classroom pedagogical use.

SitC, a collection of freely available annotated papers, aims to make primary scientific research literature more accessible to students and educators. The repository of annotated primary scientific literature articles is accessible to educators and searchable by keyword, classified by topics, and grouped in collections. The process of reading and deconstructing scientific literature in undergraduate courses has been shown to result in students potentially gaining an understanding of scientific practices, such as how scientists design their experiments and present their results, essentially allowing students to experience the logic behind drawing conclusions from a set of data [6,7,12–14].

**Fig 1:**
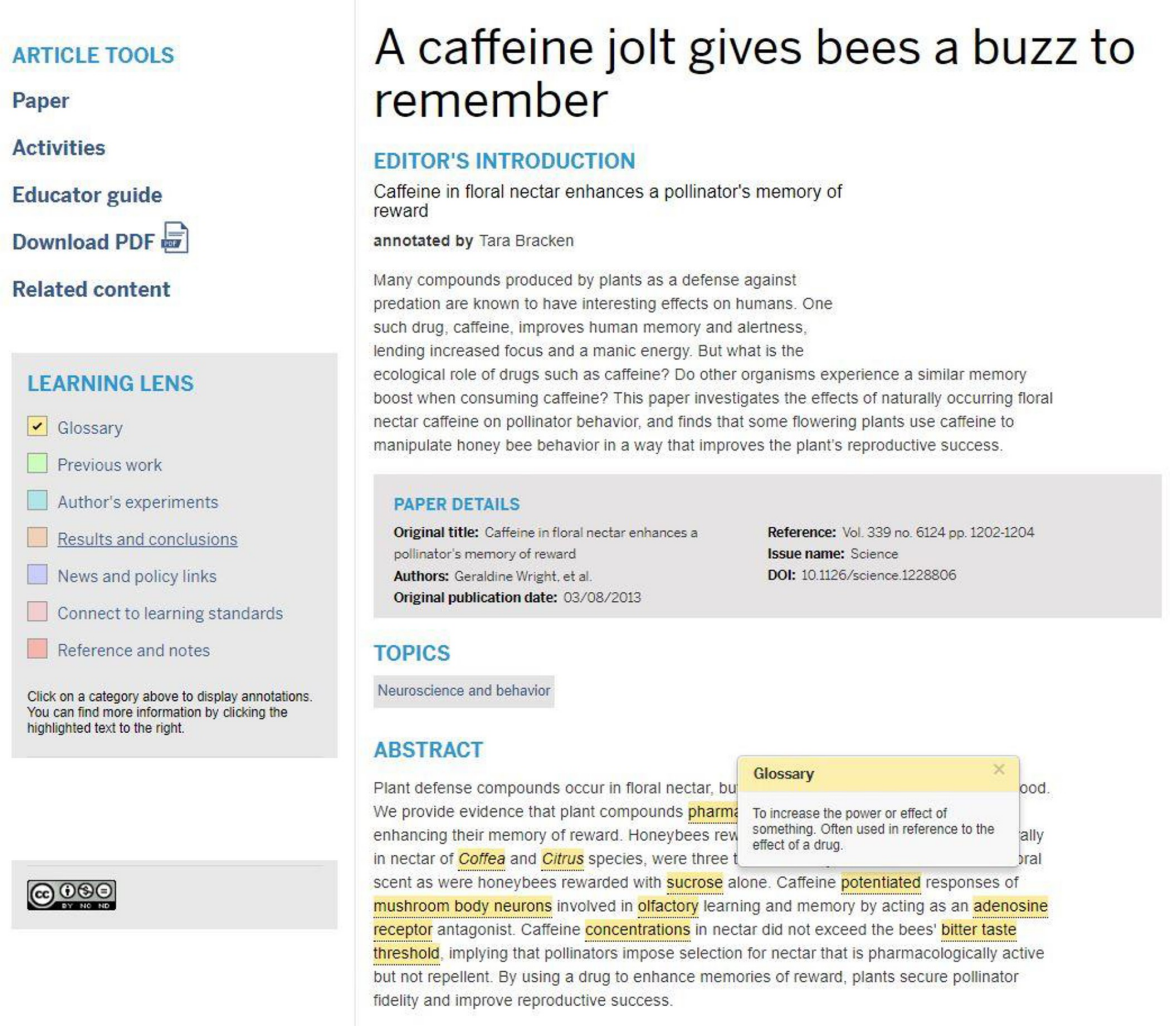
Annotated scientific research journal article used in initial implementations showing “Learning Lens” and annotations.

Annotated primary scientific literature uses the original text of research articles along with a “Learning Lens” overlay, designed to provide students tools to use for interpretation. The “Learning Lens” is used to selectively highlight different parts of the text and is composed of seven headings: Glossary, Previous work, Author's experiments, Conclusions, News and policy links, Connect to learning standards, and References and notes, which are color-coded to match the corresponding text of the research article. For example, an annotated glossary term, when clicked on, will produce a pop-up box containing the definition of the word (Fig 1). Annotations contained within the “Learning Lens” have been designed to be at the reading comprehension level of a first-year undergraduate student, and ongoing evaluation efforts have provided evidence that this goal is being met [21].

### Annotated primary literature as a pedagogical tool

Annotations provide an educational scaffold that could help students become more comfortable with reading scientific papers. We propose annotated primary scientific literature as an example of a resource that can be incorporated into existing courses and provide scaffolding that may help undergraduate students develop skills necessary to read primary scientific literature while requiring a minimal time investment from instructors. Using annotated primary scientific literature as a pedagogical tool not only could potentially help universities develop scientifically literate graduates, but it may also broaden the impact of primary scientific research literature produced by faculty.

The previously mentioned pedagogical tools and curriculum transformations can require a substantial investment of time and effort from the university, faculty, and staff. Therefore, additional tools and opportunities should be considered in order to achieve a wider variety of complementary opportunities for teaching with authentic scientific practices and engaging students in reading primary scientific literature [22]. We hypothesize that the incorporation of annotated primary scientific literature in the classroom represents one of these opportunities.

In this pilot study, we had a goal of developing an implementation protocol that could incorporate annotated primary scientific literature into undergraduate courses with a minimal time investment for instructors and minimal disruption and alteration to existing courses and plans of study.

### Implementation of annotated primary scientific literature

All data were collected in accordance with an approved FIU Institutional Review Board protocol #17–0398 and #17–0105. Our initial attempts to develop an implementation protocol for using annotated primary scientific literature as a pedagogical tool had the educational goal of introducing students to the “Learning Lens” annotations and observing how instructors and students used the tool. Initial attempts to incorporate annotated primary scientific literature focused on undergraduate biology courses at FIU, including General Biology II, Ecology, and Plant Life History. The implementation sessions were run iteratively during the same semester, ensuring that students did not overlap, and each class had only one implementation session. We describe two variations of our implementations here.

Students involved in the study self-reported their major, with 76% being biology majors. We did not collect any data on students’ prior knowledge of biology, but the majority of students in these classes are first- or second-year students.

We used the same annotated piece of primary scientific literature for all in-class activities described in this study: “Caffeine in floral nectar enhances a pollinator's memory of reward” (https://tinyurl.com/k7m329g). We chose an article that incorporated many different aspects of biology, including evolution, ecosystem interactions, basic botany, learning and memory, and animal behavior in a single study, making this paper applicable in a wide variety of undergraduate courses.

The objectives were to introduce undergraduate students to annotated primary scientific literature and collect baseline data on how students interacted with the annotations themselves. The first implementation involved a one-time intervention, connected to the student’s coursework, conducted by the researchers and began with an approximately 5-minute orientation to annotated primary scientific literature. This orientation included how to use the “Learning Lens” and a brief overview of the importance of primary scientific literature. Students were then given 20 minutes to read the selected piece of annotated primary scientific literature. At the 20-minute time point, a Qualtrics (online survey software; Provo, Utah and Seattle, Washington) link was provided, and if they were done reading, students could begin answering the feedback questionnaire. Students were given an additional 20 minutes to complete the questionnaire. Collecting and analyzing this first round of pilot data allowed for reflection on opportunities for iterative improvement.

In addition to the questionnaire data, feedback was collected through in-class activity observations conducted by the researchers. We kept detailed field notes indicating when students appeared on task, i.e., independently interacting with annotated primary scientific literature. We also noted when alternative tasks were observed, i.e., students checking email or social media, and when task completion appeared to have occurred. During the implementation, our in-class observations estimated an average time on task, i.e., interacting with annotated primary scientific literature, to be 10 minutes, because there was a noticeable increase in classroom noise after this time point. We confirmed this by using Adobe Analytics (Adobe, San Jose, California), which measures the time spent on a website by each user. We measured an average time spent on annotated primary scientific literature of 13 minutes. Due to limitations of Adobe Analytics, we are unable to collect individual data points and were limited to an aggregate average for the entire class. Note that the difference between the observed time spent on the activity and the digital measure can be explained by Adobe Analytics averaging all participants’ time spent on the article page.

The main student feedback was collected through a questionnaire containing both quantitative (content questions) and qualitative items (i.e., “what did you like about this activity?”). One of the key ideas we garnered from the qualitative data was that a one-time intervention was perceived by students as somewhat discordant when a connection between the article they read and the content they were covering at the time in their course was not made explicit by their course instructor (Table 1).

**Table 1:** Example of responses to item “Did the topic of this paper connect to your course?” from first implementation iteration.

Student	Response to item “Did the topic of this paper connect to your course?”
Example 1	No, we[‘re] studying plants.
Example 2	No, only slightly relevant to the course.
Example 3	Not necessarily because this paper was speaking about the effects of drugs on memory.
Example 4	I’m not sure.

When asked if the topic of the paper related to their course, students in this iteration gave feedback such as this activity was “only slightly relevant to the course,” and “no, we[‘re] studying plants” despite the article being explicitly about caffeine production by plants in order to attract pollinators. Additionally, we were uncertain that we had connected with the students as researchers in the same way as the instructor with whom the students had built a relationship.

Although some students may have not perceived a connection between the article content and their course content, in general, students found the annotations useful, especially regarding graphs and vocabulary interpretation. Examples of student responses can be seen in Table 2.

**Table 2:** Examples of responses to item “What did you like about the annotated paper?” from first implementation iteration.

Student	Response to item “What did you like about the annotated paper?”
Example 1	The glossary.
Example 2	Vocab definitions.
Example 3	What I mostly liked about the annotated paper was the part on how the graph had an overview, the nuts and bolts part because it is breaking down the graph for you.
Example 4	I liked the definitions being highlighted and easily defined is needed. I also liked the explanations of the graphs.

**Fig 2:**
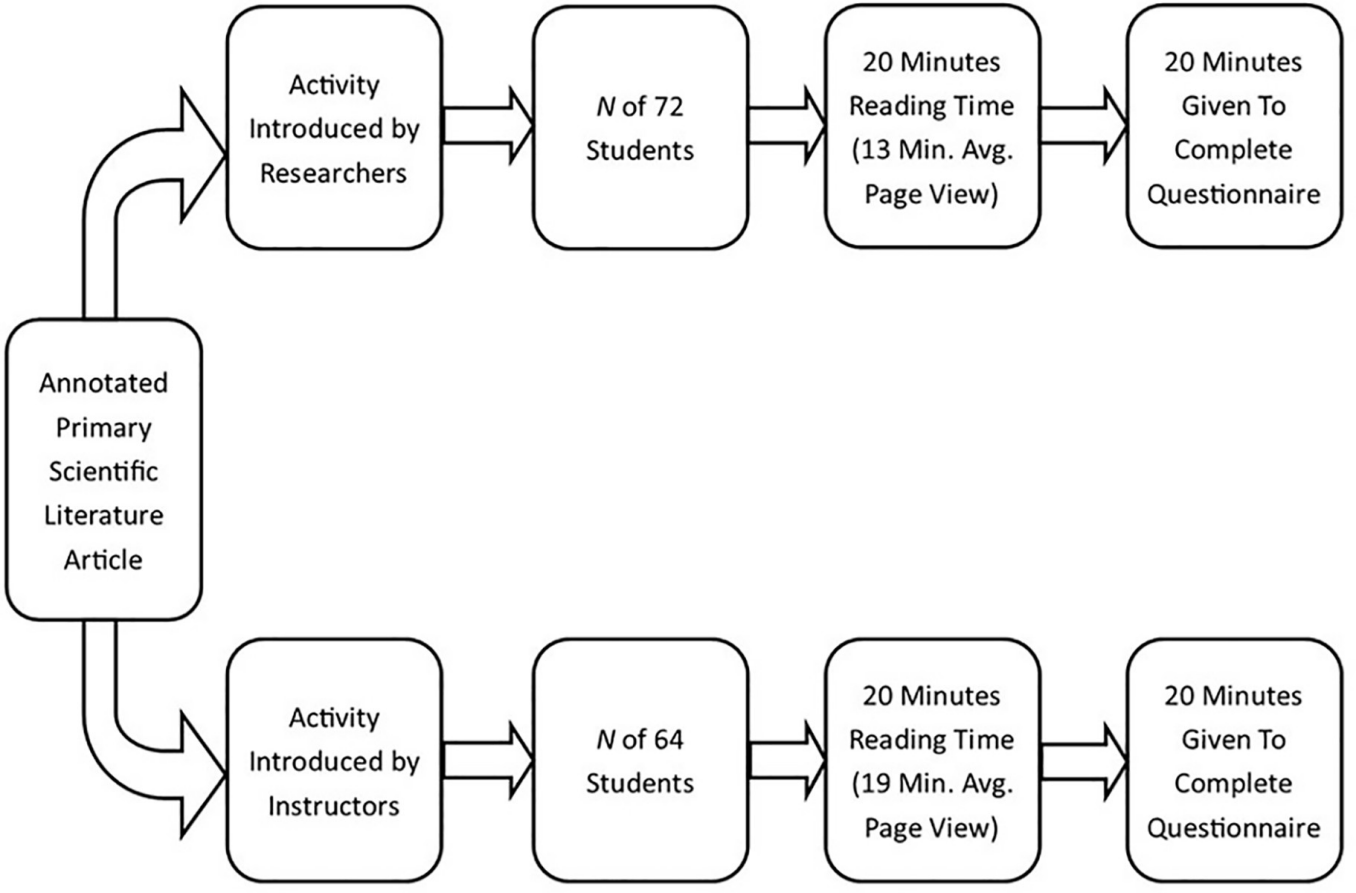
Annotated primary scientific literature implementation protocol evolution.

For our second iteration, we decided to address the issues of students feeling discordant by having the course instructors introduce the article and annotated primary scientific literature activity themselves. Additionally, we asked instructors to explicitly connect the annotated paper to current course content. With both of these procedures in place, the average time students engaged with the annotated article, as measured by Adobe Analytics, increased to 19 minutes (Fig 2).

This new implementation, in which the instructor introduced the piece of annotated primary scientific literature and annotated primary scientific literature activity, not only appeared to increase the time that students engaged with the material, but it also removed the manpower requirement for the researchers to be present in every classroom in order to describe and implement the activity. This could allow for a more widespread implementation of annotated primary scientific literature as a pedagogical tool. It was also apparent that students introduced to the activity by their course instructor were more readily able to recognize the connections between reading primary scientific research literature and their course content, which can be seen in student responses in Table 3.

**Table 3:** Example of responses to item “Did the topic of this paper connect to your course?” from second implementation iteration.

Student	Response to item “Did the topic of this paper connect to your course?”
Example 1	This article related to 3 different courses I am taking this semester.
Example 2	Yes it most certainly did.
Example 3	Yes! We’re learning about pollination.
Example 4	…scientific papers on new experiments…are important.

When asked if the topic of the paper related to their course, students in this iteration stated “This article related to 3 different courses I am taking this semester,” “yes it most certainly did,” “yes! We’re learning about pollination,” and that “…scientific papers on new experiments …are important.”

During the initial iterations of the implementation protocol, students read the annotated articles and completed an assessment during class time. However, a growing concern was feasibility of an in-class assignment due to the time requirement and allowing for instructor flexibility in scheduling. While observing a senior lecturer at FIU, who was not involved in this current study, and his existing implementation method of students reading primary scientific literature as homework and answering iClicker questions at the beginning of the following class, the researchers noticed an increased enthusiasm among the students during the class discussion. Supporting this observation, the history of research on the use of clickers in the classroom shows an increase in feelings of class involvement [23] and learning gains in students [24]. Because of the observations and support from instructors, the decision was made to adopt the homework protocol moving forward with future implementations. The homework protocol allows for more instructor freedom in selecting articles relevant to course content, reduces the class time required for implementation, and separates content questions from a pre–post attitude and motivation questionnaire. Using articles as homework also allows for instructors to utilize as many articles as they wish, but for this project moving forward, in future implementations, we will require a minimum of three articles over the course of a semester. We are currently piloting an implementation protocol using annotated primary scientific literature as a homework assignment and are excited to see how instructors and students use annotated primary scientific literature moving forward.

### Advice to others

In the ongoing iterative development of an implementation protocol for annotated primary scientific literature, the most fruitful exercise has been reflection. This is great practice for any educator or educational researcher during the curriculum or pedagogical tool development process. Reflection on early classroom implementations helped us identify the opportunities for improvement in our subsequent protocol iterations and allowed us to make modifications based upon quantitative, qualitative, and observational data. One example of changes coming from reflection was noticing that during an implementation, students were opening the assessment without reading the article and using the “find” feature within the article to find answers to assessment questions. This led to preventing entry into the assessment until the time for reading had elapsed. Our subsequent classroom observations showed us that this forced students to interact with the article and be more thoughtful about their answers to the assessment, i.e., answers were not cut-and-pasted from the article text. We advise others to continue this practice of thoughtful reflection when using annotated primary scientific literature as a pedagogical tool. We also welcome any feedback or alternative uses of annotated primary scientific literature.

### Future steps

The latest annotated primary scientific literature implementation protocol iteration is being pilot tested during fall 2018. Focusing more on robust evaluation now that implementation obstacles have been overcome will allow us to determine the effectiveness of annotated primary scientific literature as a pedagogical tool in undergraduate biology classrooms. Future studies are being designed to examine students’ scientific literacy before and after completing the annotated article activities using a previously validated scientific literacy instrument (Test of Scientific Literacy Skills [TOSLS]) [2]. Additionally, we aim to measure students’ subjective task values with regards to reading primary scientific research literature [25–28], as well as their primary scientific literature reading self-efficacy [29–32].

We hope to spread the word about annotated primary scientific literature and investigate its potential impacts on student learning and motivation as we further refine our implementation protocol and propagate beyond our department and institution.

## Abbreviations

CREATE: consider, read, elucidate the hypotheses, Analyze and interpret the data, and think of the next experiment
FIU: Florida International University
SitC: Science in the Classroom
STEM: science, technology, engineering, and math
TOSLS: Test of Scientific Literacy Skills
